# Transvaginal Ultrasound-Guided Thrombin Injection for Post-oocyte Retrieval Pseudoaneurysm in Pregnancy

**DOI:** 10.7759/cureus.84159

**Published:** 2025-05-15

**Authors:** Vishnu Prasad Pulappadi, Santhosh Poyyamoli, Sumathi Natarajan, Pankaj Mehta, Mathew Cherian

**Affiliations:** 1 Interventional Radiology, Kovai Medical Center and Hospital, Coimbatore, IND; 2 Fetal Radiology, Kovai Medical Center and Hospital, Coimbatore, IND

**Keywords:** embolization, in vitro fertilization, pregnancy, pseudoaneurysm, ultrasound

## Abstract

Transvaginal sonography (TVS)-guided oocyte retrieval is an assisted reproductive technology commonly performed for in vitro fertilization. This report presents the case of a 42-year-old primigravida, who presented in the second trimester of pregnancy with vaginal bleeding and left iliac fossa pain. Magnetic resonance imaging and TVS revealed a pseudoaneurysm in the left parametrium. To avoid radiation exposure to the fetus, TVS-guided embolization was planned for the patient. Under TVS guidance, a Chiba needle was advanced into the pseudoaneurysm sac, and lyophilized thrombin powder reconstituted in calcium chloride solution was injected. Immediate thrombosis of the pseudoaneurysm was observed. Her abdominal pain reduced after the procedure, and she did not have any further episodes of vaginal bleeding. Follow-up TVS performed after one month showed persistent occlusion of the pseudoaneurysm.

## Introduction

The use of assisted reproductive technology has increased over the past few decades. The most commonly used technology is in vitro fertilization (IVF), which involves oocyte retrieval, fertilization, and embryo transfer. Oocyte retrieval is now commonly performed transvaginally under ultrasonography (USG) guidance [1]. Although minimally invasive, transvaginal oocyte retrieval can rarely result in vascular injury when the pelvic blood vessels are traversed during needle insertion [2-4]. Pseudoaneurysm, a contained rupture of the blood vessel, may develop following vascular injury. Due to the high risk of rupture, urgent treatment is indicated for all pseudoaneurysms. They are best treated by endovascular embolization using coils or liquid embolic agents. However, transarterial embolization of pelvic pseudoaneurysms in pregnant women is challenging because special care needs to be taken to avoid radiation exposure to the fetus, which can lead to miscarriage and congenital malformation, and ischemia to the uterus.

Direct percutaneous access followed by embolization is an alternative treatment option for pseudoaneurysms when transarterial embolization is unsuccessful or not feasible [5]. It is also done for superficially located pseudoaneurysms, such as iatrogenic femoral artery pseudoaneurysms that develop after percutaneous access for transarterial interventions [6]. Thrombin and n-butyl cyanoacrylate glue are the most commonly used agents for percutaneous embolization. Injection of thrombin into the pseudoaneurysm sac activates fibrinogen, resulting in thrombus formation. It is more likely to be successful in small pseudoaneurysms [7].

Ultrasound-guided embolization of pelvic pseudoaneurysm in pregnancy has yet to be reported in the literature. This report presents a case of iatrogenic pseudoaneurysm complicating an IVF-assisted pregnancy, treated by transvaginal sonography (TVS)-guided thrombin injection.

## Case presentation

A 42-year-old primigravida presented at 16 weeks and five days of gestation with left iliac fossa pain radiating to the back for one month. She had minimal vaginal bleeding one week before the current presentation. She was hypothyroid and underwent a laparoscopic myomectomy four years ago. Her conception was by IVF performed at another hospital. TVS-guided oocyte retrieval was done, followed by intracytoplasmic sperm injection and embryo transfer. She had a triplet pregnancy and underwent reduction of one of the fetuses at the 13th week of gestation. The reduction of the fetus located near the uterine fundus was done using transabdominal USG guidance. Her abdominal pain started two days after the fetal reduction. At presentation, her hemodynamic vital parameters were stable.

**Figure 1:**
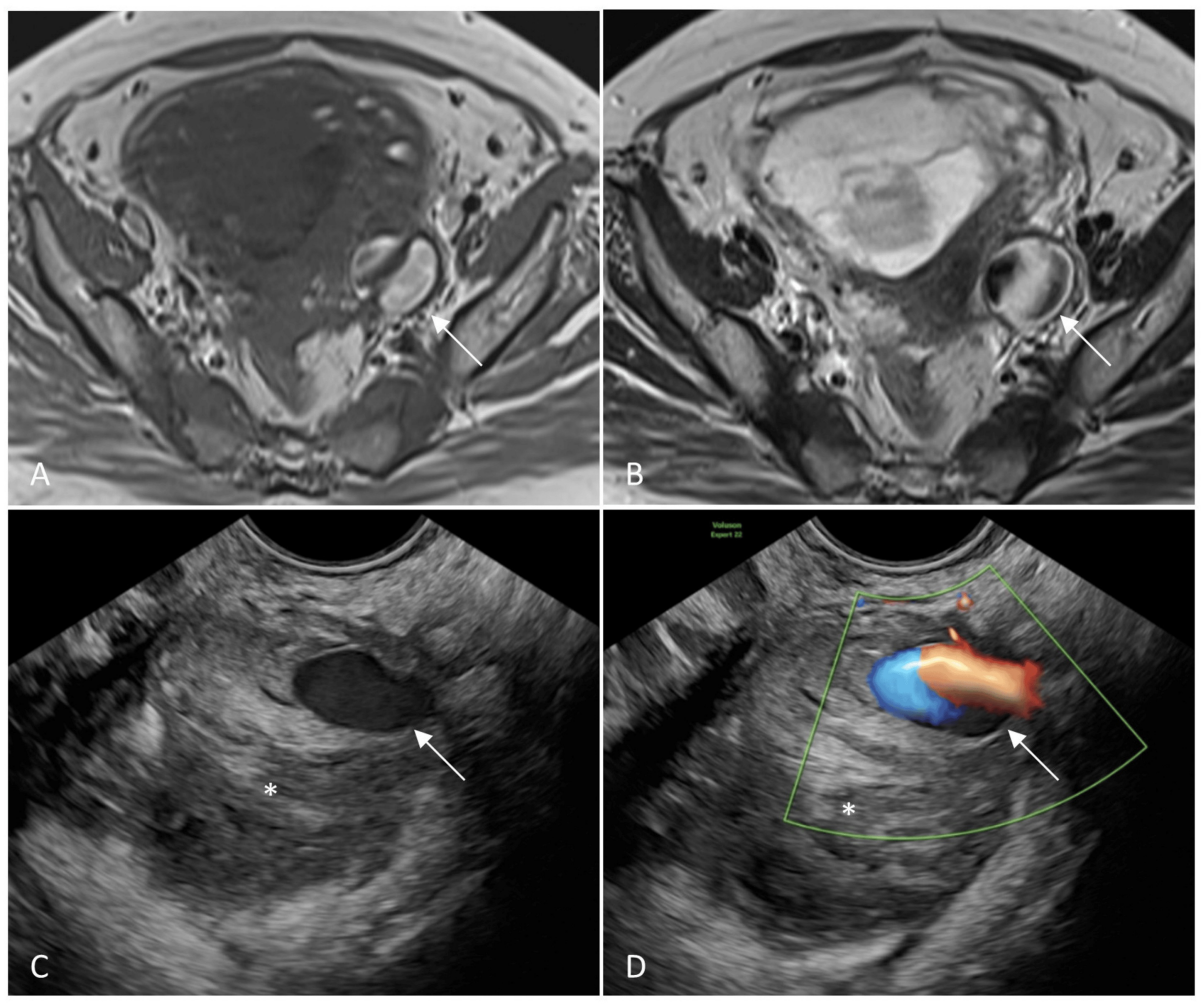
Pseudoaneurysm in the left parametrium Axial (A) T1- and (B) T2-weighted magnetic resonance images showing a well-defined T1 and T2 hyperintense lesion in the left parametrium, with flow voids along the periphery of the lesion (arrows). (C) B-mode and (D) color Doppler transvaginal ultrasonography images showing an anechoic lesion (arrow in D) with a yin-yang flow pattern (arrow in D), suggestive of a pseudoaneurysm. Echogenic hematoma was seen around the pseudoaneurysm (asterisk)

Imaging evaluation was done at another hospital to rule out the common causes of abdominal pain, like miscarriage, ectopic pregnancy, and ovarian torsion. Transabdominal USG failed to reveal any abnormality. Magnetic resonance imaging (MRI) of the abdomen and the pelvis was done to ascertain the cause of the pain. It showed a T1 and T2 hyperintense lesion in the left parametrium, measuring 3.7×3.3 cm, with flow voids along its periphery (Figure 1A, 1B). The patient presented to our center one week after the MRI. TVS was performed, and it confirmed the presence of a pseudoaneurysm in the left parametrium adjoining the vaginal wall, measuring 2.5×1 cm in size, with a hematoma measuring 4.5×4.1 cm surrounding it (Figure 1C, 1D). The artery of origin of the pseudoaneurysm could not be identified on TVS. As the pseudoaneurysm was easily accessible through the transvaginal route and transarterial embolization had the risk of radiation exposure to the fetus, it was decided to proceed with ultrasound-guided direct transvaginal embolization of the pseudoaneurysm. Thrombin was chosen as the embolizing agent because of its high efficacy, rapid action, and low migration risk.

**Figure 2:**
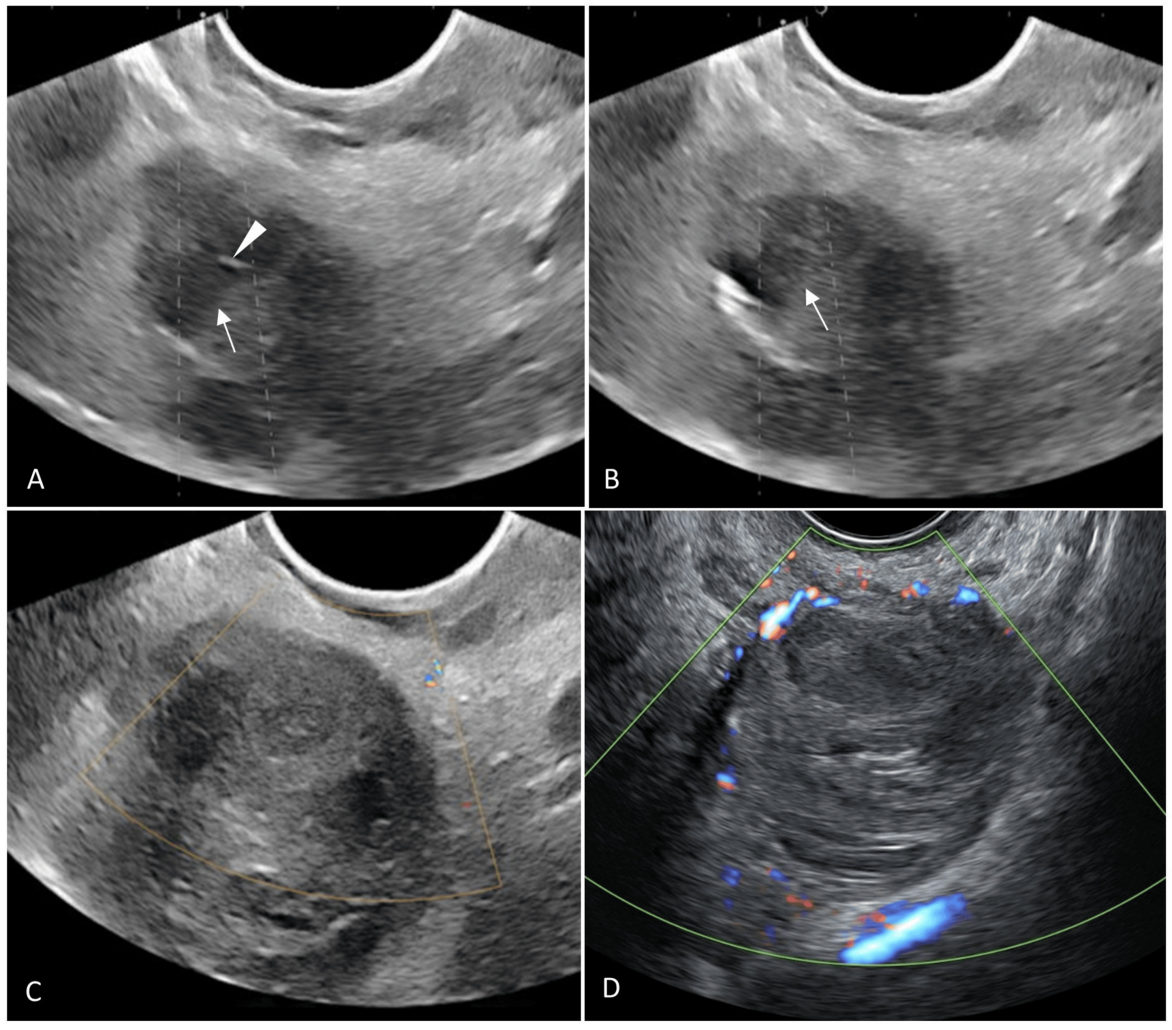
TVS-guided thrombin injection (A) TVS image showing a 22G Chiba needle (arrowhead) being advanced into the pseudoaneurysm (arrow). (B) TVS image showing echogenic thrombus (arrow) formed within the pseudoaneurysm following thrombin injection. (C) Post-thrombin injection color Doppler TVS showing no residual filling of the pseudoaneurysm. (D) Follow-up color Doppler TVS performed after one month showing persistent occlusion of the pseudoaneurysm with reduction in the size of the hematoma TVS: transvaginal sonography

The procedure was performed under spinal anesthesia, and TVS was done on an Aixplorer USG system (Supersonic Imagine, Aix-en-Provence, Auvergne-Rhône-Alpes, France) using a 3-12 MHz endocavity transducer. Lyophilized thrombin powder (500 U) (Tisseel Lyo, Baxter AG, Vienna, Austria) was reconstituted in 1 ml calcium chloride solution and pre-heated to 37°C on the Fibrinotherm device (Baxter AG, Vienna, Austria). Under TVS guidance, a 22G 20-cm-long Chiba needle (Cook Medical, Bloomington, Indiana, United States) was advanced into the pseudoaneurysm (Figure 2A). Once the position was confirmed by the backflow of blood after the removal of the stylet, a three-way stopcock was attached to the hub of the needle. We planned to inject the thrombin solution in aliquots of 0.2 ml each, with monitoring of fetal cardiac activity on USG after each injection. Following a saline flush to purge out the blood from the lumen of the needle, 0.2 ml of thrombin solution (100 U) was injected into the pseudoaneurysm under USG guidance. This was followed by the injection of 2 ml of normal saline. Instantaneous thrombus formation was observed on USG (Figure 2B). Color Doppler USG showed complete thrombosis of the pseudoaneurysm (Figure 2C). Transabdominal USG confirmed normal cardiac activity in both fetuses. No peri-procedural complications were observed. The patient had no further episodes of vaginal bleeding, and her abdominal pain gradually reduced in severity. Follow-up TVS done after one month showed a small residual hematoma measuring 3.1×3 cm, with a completely occluded pseudoaneurysm (Figure 2D). Transabdominal USG showed normal growth parameters in both fetuses.

## Discussion

Uterine artery and vaginal wall pseudoaneurysms can develop following assisted vaginal delivery, cesarean section, transabdominal uterine surgeries, and transvaginal interventions such as curettage, intra-uterine contraceptive device insertion, and brachytherapy [8-11]. They are often detected on USG as it is the first-line imaging modality used for the evaluation of patients presenting with vaginal bleeding. TVS is superior to transabdominal USG because of its excellent image quality and better visualization of the vaginal wall and the adnexa. A pseudoaneurysm is seen on USG as a well-defined anechoic lesion with a characteristic "yin-yang" sign on color Doppler due to turbulent blood flow. In the current case, the pseudoaneurysm was not detected on the initial transabdominal USG. However, the presence of flow voids within the lesion on T2-weighted MR images helped in making a diagnosis.

TVS-guided oocyte retrieval has become increasingly popular. Although transient vaginal bleeding may occur after oocyte retrieval, persistent bleeding or hemodynamic instability should raise the suspicion of an arterial injury. Injury may occur to the uterine artery, branches of the internal pudendal artery supplying the vaginal wall, or obturator artery located along the pelvic side wall [2-4]. Because an angiography was not performed, the artery of origin of the pseudoaneurysm could not be ascertained in the present case. Although the patient in the present case had a history of myomectomy and transabdominal USG-guided fetal reduction, the superficial location of the pseudoaneurysm in the parametrium adjoining the vaginal wall indicates that it would have developed as a result of vascular injury during oocyte retrieval through the TVS route. The patient in the present case was initially asymptomatic and presented in the second trimester of pregnancy with vaginal bleeding and abdominal pain. Delayed presentation of post-oocyte retrieval pseudoaneurysm in pregnancy has been reported previously [3]. During pregnancy, the circulating blood volume and cardiac output increase, resulting in hyperdynamic circulation. As a result, undetected small pseudoaneurysms may enlarge and rupture later in pregnancy.

Although spontaneous thrombosis of small asymptomatic uterine artery pseudoaneurysms has been reported [12], urgent treatment is indicated for all pseudoaneurysms to prevent life-threatening hemorrhage. Transarterial embolization has been described for post-oocyte retrieval uterine artery pseudoaneurysms detected during pregnancy [3]. However, because of the direct X-ray exposure to the pelvis and the need for oblique projections for super-selective catheterization of the culprit artery, there is a risk of high radiation exposure to the fetus. In addition, transarterial embolization of uterine artery pseudoaneurysms carries the risk of ischemia to the placenta and fetus. On the other hand, USG-guided embolization eliminates the need for radiation exposure and reduces the risk of ischemia to the uterus as the embolizing agent is directly injected into the pseudoaneurysm. Therefore, it can be done as the first-line treatment for pelvic pseudoaneurysms in pregnancy whenever feasible. It is commonly done to treat femoral artery pseudoaneurysms with a high treatment success rate of 86% [7]. There are a few case reports of TVS-guided thrombin injection performed as the first-line treatment for iatrogenic pelvic pseudoaneurysms [10,13,14]. It can also be done as a rescue treatment for myometrial pseudoaneurysms that fail to occlude after uterine artery embolization due to collateral supply from the ovarian arteries [15]. To the best of our knowledge, the current case is the first one reported in the literature of a pelvic pseudoaneurysm in pregnancy treated by TVS-guided thrombin injection. Thrombin is classified as a Category C drug due to the lack of human studies. The present case did not have a recurrence of pseudoaneurysm on the follow-up imaging done after one month. One case of recurrence has been reported in the literature, in which there was recurrent bleeding from a uterine artery pseudoaneurysm after thrombin injection, requiring transarterial embolization [14].

## Conclusions

Vascular injury and pseudoaneurysm formation can rarely occur following transvaginal interventions. Pseudoaneurysms developing after transvaginal oocyte retrieval may stay asymptomatic for many weeks before manifesting with vaginal bleeding in pregnancy. Prompt detection using TVS helps in providing timely treatment for such patients. The risk of life-threatening hemorrhage necessitates urgent embolization for all pseudoaneurysms. Although transarterial embolization is the standard of care for pseudoaneurysms, it requires angiography and involves radiation exposure. TVS-guided thrombin injection is a simple and effective treatment for pelvic pseudoaneurysms that are accessible by the transvaginal route. Because of the advantage of avoiding radiation exposure to the fetus and minimizing the risk of uterine ischemia, it has a potential role as the first-line treatment for superficially located pelvic pseudoaneurysms in pregnancy. Further studies are needed to evaluate its long-term efficacy and safety and the risk of pseudoaneurysm recurrence.
